# Epigenomic and functional analyses reveal roles of epialleles in the loss of photoperiod sensitivity during domestication of allotetraploid cottons

**DOI:** 10.1186/s13059-017-1229-8

**Published:** 2017-05-31

**Authors:** Qingxin Song, Tianzhen Zhang, David M. Stelly, Z. Jeffrey Chen

**Affiliations:** 1 Department of Molecular Biosciences, Institute for Cellular and Molecular Biology, and Center for Computational Biology and Bioinformatics, The University of Texas at Austin, Austin, TX 78712 USA; 2 State Key Laboratory of Crop Genetics and Germplasm Enhancement, Nanjing Agricultural University, Nanjing, 210095 China; 3 Department of Soil and Crop Sciences, Texas A&M University, College Station, TX 78743 USA

**Keywords:** DNA methylation, Epigenomics, Cotton, Photoperiod, Hybrid, Polyploidy, Biotechnology

## Abstract

**Background:**

Polyploidy is a pervasive evolutionary feature of all flowering plants and some animals, leading to genetic and epigenetic changes that affect gene expression and morphology. DNA methylation changes can produce meiotically stable epialleles, which are transmissible through selection and breeding. However, the relationship between DNA methylation and polyploid plant domestication remains elusive.

**Results:**

We report comprehensive epigenomic and functional analyses, including ~12 million differentially methylated cytosines in domesticated allotetraploid cottons and their tetraploid and diploid relatives. Methylated genes evolve faster than unmethylated genes; DNA methylation changes between homoeologous loci are associated with homoeolog-expression bias in the allotetraploids. Significantly, methylation changes induced in the interspecific hybrids are largely maintained in the allotetraploids. Among 519 differentially methylated genes identified between wild and cultivated cottons, some contribute to domestication traits, including flowering time and seed dormancy. *CONSTANS* (*CO*) and *CO*-*LIKE* (*COL*) genes regulate photoperiodicity in *Arabidopsis. COL2* is an epiallele in allotetraploid cottons. *COL2A* is hypermethylated and silenced, while *COL2D* is repressed in wild cottons but highly expressed due to methylation loss in all domesticated cottons tested. Inhibiting DNA methylation activates *COL2* expression, and repressing *COL2* in cultivated cotton delays flowering.

**Conclusions:**

We uncover epigenomic signatures of domestication traits during cotton evolution. Demethylation of *COL2* increases its expression, inducing photoperiodic flowering, which could have contributed to the suitability of cotton for cultivation worldwide. These resources should facilitate epigenetic engineering, breeding, and improvement of polyploid crops.

**Electronic supplementary material:**

The online version of this article (doi:10.1186/s13059-017-1229-8) contains supplementary material, which is available to authorized users.

## Background

Polyploidy or whole genome duplication (WGD) is a pervasive evolutionary feature of some animals and all flowering plants [1, 2], leading to genetic and epigenetic changes that affect gene expression and morphology [3–5]. Estimates indicate that two rounds of ancestral WGD occurred before the divergence of extant seed plants and angiosperms, giving rise to the diversification of genes and pathways important to seed and flower development and eventually the dominance of angiosperms on the earth [6, 7]. Many important crops including wheat, cotton, and canola are allopolyploids, which usually arise via fusion of 2*n* gametes between species or by interspecific hybridization followed by genome doubling [3, 8]. Genomic interactions in the polyploids can induce genetic and epigenetic changes including DNA methylation [1, 3]. DNA methylation changes can produce meiotically stable epialleles [9, 10] which are transmissible through natural selection and breeding. For example, stable DNA methylation in promoters can be inherited as epialleles, which confer symmetric flower development in *Linaria vulgaris* [11] and quantitative trait loci of colorless non-ripening and vitamin E content in tomato [12, 13]. In plants, DNA methylation occurs in CG, CHG, and CHH (H = A, T, or C) contexts through distinct pathways [14]. In *Arabidopsis*, maintenance methylation of CG and CHG is regulated by *METHYLTRANSFERASE1* (*MET1*) and *CHROMOMETHYLASE3* (*CMT3*), respectively [15–17]. De novo CHH methylation is established by RNA-directed DNA methylation (RdDM) and CMT2-mediated pathways [18–20]. DNA methylation is essential for maintaining animal and plant development. Methylation defects are embryonic lethal in animals [21], induce additional epigenetic changes during self-pollination in *Arabidopsis* [22], and lead to lethality in rice [23]. DNA methylation is also responsible for seed development [24] and adaptation to environments [25]. Furthermore, DNA methylation changes are associated with expression of homoeologous genes in resynthesized and natural *Arabidopsis* allotetraploids [26–28], natural *Spartina* allopolyploids [29], and paleopolyploid beans [30]. However, epigenomic resources in polyploids are very limited, and the functional role of epialleles in morphological evolution and crop domestication remains largely unknown.

**Fig. 1:**
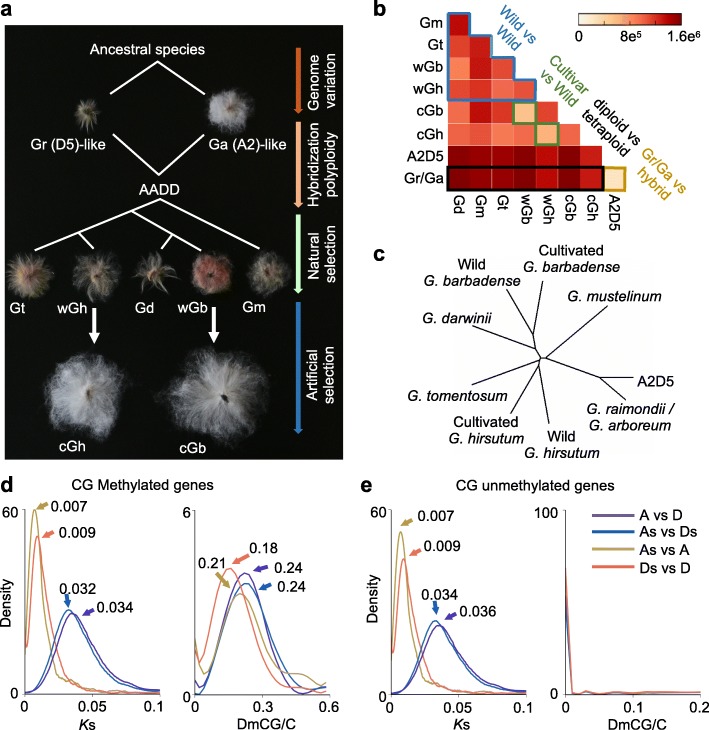
Evolution of DNA methylation and genome sequence during polyploidization in cotton. **a** Allotetraploid cotton (*AADD*) was formed between A-genome species like *G. arboreum* (*Ga*) and D-genome species like *G. raimondii* (*Gr*), giving rise to five allotetraploid species: wild *G. hirsutum* (*wGh*), wild *G. barbadense* (*wGb*), *G. tomentosum* (*Gt*), *G. darwinii* (*Gd*), and *G. mustelinum* (*Gm*). Wild Gh and Gb are domesticated into cultivated *G. hirsutum* (*cGh*) and *G. barbadense* (*cGb*), respectively. **b** The number of differentially methylated cytosines in CG context (*DmCG*) in each pairwise comparison between different cotton species as shown in **a**. *A2D5* is an interspecific hybrid between Ga (A2) and Gr (D5). *Blue*, *green*, *black*, and *yellow* brackets indicate comparisons of wild vs. wild, cultivated vs. wild allotetraploids, diploid vs. allotetraploid, and diploid parents vs. interspecific hybrid, respectively. **c** Phylogenetic tree was reconstructed based on genome-wide mCG divergent levels among cotton species. **d**, **e** Distribution of synonymous substitution values (*K*s) (*left*) and gene-body DmCG percentages (*right*) of 6781 methylated orthologous genes (**d**) and 4063 unmethylated orthologous genes (**e**). *As*, *Ds* A subgenome and D subgenome in cultivated *G. hirsutum*, *A G. arboreum*, *D G. raimondii*. Peak values are indicated by *arrows*. The rate of methylation changes in each gene pair was estimated as the number of DmCG divided by the total number of CG in the gene body

Cotton is the largest source of renewable textile fiber and an excellent model for studying polyploid evolution and crop domestication [31, 32]. Allotetraploid cotton was formed approximately 1– 1.5 million years ago (MYA) [33] by interspecific hybridization between two diploid species, one having the A genome like in *Gossypium arboreum* (Ga, A2) and *Gossypium herbaceum* (A1), and the other resembling the D5 genome found in extant species *Gossypium raimondii* (Gr); divergence of A-genome and D-genome ancestors is estimated at ~6 MYA (Fig. 1a). The allotetraploid diverged into five or more species [32, 34]. Two of them, *Gossypium hirsutum* (Gh, Upland cotton) and *Gossypium barbadense* (Gb, Pima cotton), were independently domesticated for higher fiber yield and wider geographical distribution; these characteristics were accompanied by extraordinary morphological changes including loss of photoperiod sensitivity, reduction in seed dormancy, and conversion from tree-like wild species to an annual crop [31, 33, 35].

Here we generated single-base resolution methylomes of domesticated allotetraploid cottons and their wild tetraploid and diploid relatives. More than 12 million differentially methylated cytosines across all species were comparatively analyzed, which revealed different rates of DNA methylation and sequence changes and distinct methylation distributions in transposable elements (TEs) and genes. Integrating the data of methylomes with transcriptomes, we discovered more than 500 putative epialleles that may contribute to morphological and physiological changes during evolution and domestication of polyploid cottons. Genomic and functional analyses of an epiallele confirmed its role in photoperiodic flowering, contributing to wider geographical distribution of cotton. We propose that epigenomic resources can be used to improve crop production by epigenetic engineering and breeding.

## Results

### Different rates between DNA methylation changes and sequence evolution

To uncover DNA methylation changes during cotton evolution and domestication, we generated single-base resolution methylomes from diploid *G. arboreum* (A2), diploid *G. raimondii* (D5), their interspecific hybrid (A2D5), wild allotetraploid *G. hirsutum* (wGh), wild allotetraploid *G. barbadense* (wGb), allotetraploid *G. tomentosum* (Gt), allotetraploid *G. mustelinum* (Gm), allotetraploid *G. darwinii* (Gd), cultivated allotetraploid *G. hirsutum* (cGh), and cultivated allotetraploid *G. barbadense* (cGb) (Fig. 1a; Additional file 1: Table S1). To exclude the effect of nucleotide variation across species (especially between C and T) on DNA methylation analysis, we identified 352,667,453 conserved cytosines (~48% of the total cytosines of the genome) between all species and present in two biological replicates for further analysis (Additional file 2: Figure S1). Among them, 12,045,718 (~3.4% of) differentially methylated cytosines (DmCs) were found across all species; there were more DmCs between diploid cottons and tetraploid cottons (diploid vs. tetraploid) than for other comparisons (diploid vs. diploid cottons, wild tetraploid vs. wild tetraploid, and wild vs. cultivated cottons) (Fig. 1b).

Methylation divergent levels at CG and non-CG sites, respectively, that were conserved among all species (Additional file 2: Figure S1) were used to generate neighbor-joining phylogenetic trees. Phylogenetic trees with CG and non-CG sites recapitulated the known evolutionary relationships of cotton species [32], including sister taxa relationships between *G. hirsutum* and *G. tomentosum* and between *G. barbadense* and *G. darwinii* (Fig. 1c; Additional file 2: Figure S2). This suggests concerted evolution between DNA sequence and methylation changes. Gene-body methylated genes occur largely in CG sites [36] and evolve slowly [37]. To test the relationship between methylation and sequence evolution in genic regions, we divided orthologous genes into CG body-methylated (*P*_CG_ < 0.05) and CG body-unmethylated genes (*P*_CG_ > 0.95) using a binomial test with body-methylation levels [37]. To reduce the effect of non-CG methylation on CG methylation analysis, CHG or CHH body-methylated orthologous genes (*P*_CHG_ < 0.05 or *P*_CHH_ < 0.05) were removed. Among CG body-methylated genes, the percentage of CG methylation changes (peaks at 0.18–0.24) was substantially higher than the substitution rate of coding sequence (*Ks* value peaks at 0.007–0.034) (Fig. 1d), suggesting that the methylation change rate is faster than the neutral sequence substitution rate. In the CG body-unmethylated genes, although the sequence variation remained at a similar level, the methylation peak disappeared (Fig. 1e).

### DNA methylation divergence between progenitor-like diploid species

**Fig. 2:**
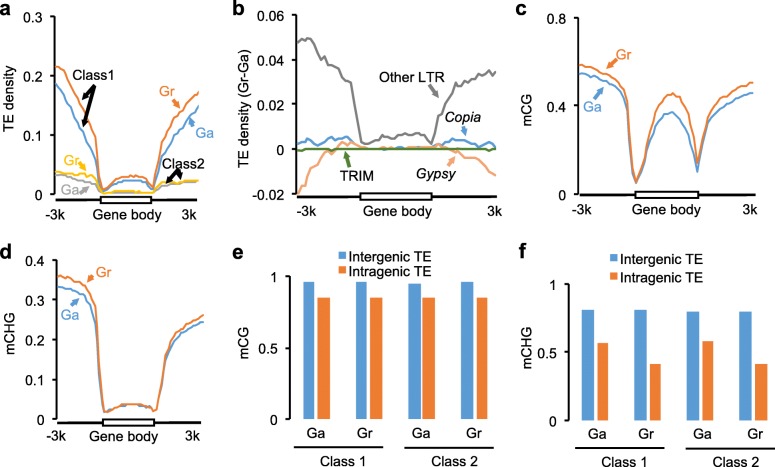
Asymmetrical distribution of TE and DNA methylation in A and D genomes. **a** Distribution of class I and II TEs in genic regions of *G. arboreum* (Ga, *blue* for I and *gray* for II) and *G. raimondii* (Gr, *orange* for I and *yellow* for II). **b** Density differences (between *G. raimondii* and *G. arboreum*) of *Gypsy* (*orange*), *Copia* (*blue*), *TRIM* (terminal repeat retrotransposons in miniature, *green*), and other long terminal repeats (*LTR*, *gray*) in the genic regions. **c**, **d** CG (**c**) and CHG (**d**) methylation levels in the genic regions of *G. arboreum* and *G. raimondii*. **e** Average CG methylation differences between intergenic and intragenic TEs. **f** Average CHG methylation differences between intergenic and intragenic TEs

TEs are often associated with DNA methylation and genome complexity [14, 38, 39]. In diploid species, the *G. arboreum* genome is twofold larger and contains more TEs than the *G. raimondii* genome, probably because of TE expansion in the centromeric and peri-centromeric regions [40, 41] (Additional file 2: Figure S3a). However, in genic regions there were more DNA TEs and especially retrotransposons in *G. raimondii* than in *G. arboreum* (Fig. 2a). For retrotransposons, *G. raimondii* had more *Copia* and unclassified long terminal repeats (LTRs) in flanking sequences of the gene body than *G. arboreum* (Fig. 2b). Terminal repeat retrotransposons in miniature (TRIMs), which are enriched near genes [42, 43], showed similar distribution patterns between *G. raimondii* and *G. arboreum* (Fig. 2b). Because of high CG methylation levels in the TEs, *G. raimondii* homoeologs were generally more methylated than *G. arboreum* homoeologs (Fig. 2c; Additional file 2: Figure S3b).

Although TEs were also associated with high CHG methylation levels (Additional file 2: Figure S3b), CHG methylation levels in the gene body were similar between *G. raimondii* and *G. arboreum* (Fig. 2d). CHG methylation correlates positively with the repressive histone mark H3K9 methylation and negatively with gene expression [19, 39]. We speculate that TEs inserted in the gene body (intragenic TEs) could gradually lose CHG methylation during evolution to prevent silencing. Consistent with the hypothesis, CHG methylation levels were lower in the intragenic TEs than in the intergenic TEs; reduction in CHG methylation (27–49%) is higher than decrease in CG methylation (~12%) (Fig. 2e, f). CHG methylation levels of intragenic TEs were decreased to lower levels in *G. raimondii* than in *G. arboreum* (Fig. 2e, f). As a result, CHG methylation levels in the gene body were similar between them, although *G. raimondii* has more TEs in the gene body than *G. arboreum* (Fig. 2b, d).

### Hybrid-induced DNA methylation changes are conserved in polyploids

**Fig. 3:**
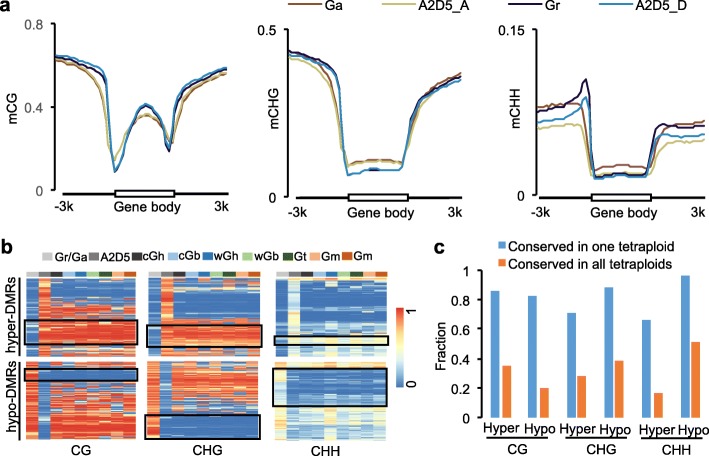
DNA methylation changes induced by interspecific hybridization were largely maintained during allotetraploid evolution. **a** CG (*left*), CHG (*middle*), and CHH (*right*) methylation changes between A2D5 hybrid and the parents (*G. arboreum* and *G. raimondii*) in gene regions. **b** Clustering analysis of differentially methylated regions (*DMRs*) between A2D5 interspecific hybrid and the parents, which were present among seven allotetraploids including two domesticated cottons. Each row indicates one DMR. Species names were abbreviated as in Fig. 1a. *Black boxes* indicate conserved DMRs that showed the same trend of DNA methylation changes in allotetraploids as in the A2D5 hybrid relative to progenitor-like diploid species (*G. arboreum* and *G. raimondii*). **c** Fraction of conserved CG, CHG, and CHH hyper or hypo DMRs in one allotetraploid (*blue*) or all allotetraploids (*orange*) shown in **b**. Absolute values of methylation change threshold for conserved DMRs were 0.4 for CG and CHG DMRs and 0.1 for CHH DMRs

The DNA methylation difference between the species could induce changes in allopolyploids [26, 27, 44]. Surprisingly, compared with the parents, CG and CHG methylation levels in the gene body were similar, but CHH methylation levels were decreased in the interspecific hybrids (A2D5) that were formed between *G. raimondii* and *G. arboreum* (Fig. 3a). This is different from *Arabidopsis* intraspecific hybrids that display higher methylation levels in all contexts than the parents [45, 46]. We further examined whether hybridization-induced methylation changes could be maintained during evolution, compared to polyploidization of allotetraploid cottons that had diverged into five or more different species [34]. To test this, we identified 44,386 CG, 17,269 CHG, and 7461 CHH differentially methylated regions (DMRs) between the interspecific hybrid (A2D5) and parents (Additional file 3: Table S2). Among them, proportions of conserved CG, CHG, and CHH DMRs were 66–96% and 17–51% in at least one and all allotetraploid species tested, respectively, which were significantly higher than random events (*P* < 1e^-200^, hypergeometric test) (Fig. 3b, c). Although the exact progenitors of ancient allotetraploids are unknown, the data suggest that a wide range of hybridization-induced DNA methylation changes is conserved during polyploid evolution.

### Role of DNA methylation in homoeologous expression bias

**Fig. 4:**
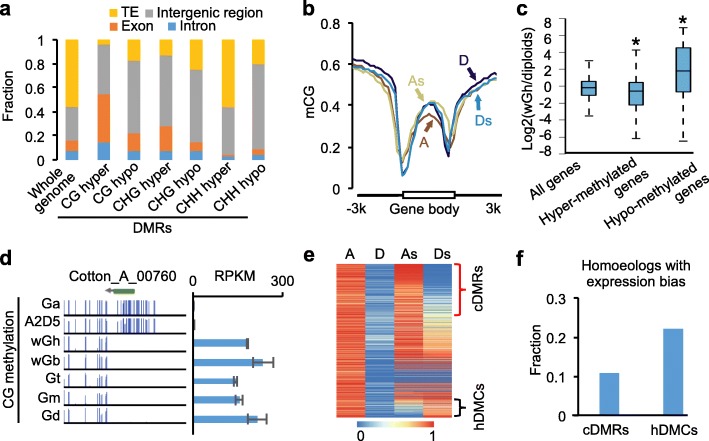
DNA methylation changes between diploid species (*G. arboreum* and *G. raimondii*) and allotetraploid cottons. **a** Fraction of CG, CHG, and CHH hyper or hypo DMRs between diploid and wild allotetraploid cottons in different genomic features. Intergenic regions indicate those located between gene bodies excluding TEs. **b** CG methylation levels of Ga (*A*), Gr (*D*), Gh A subgenome (*As*), and D subgenome (*Ds*) in genic regions. **c** Log2 expression ratios (wGh/diploids) of all genes, hypermethylated genes, and hypomethylated genes. **d** An example of CG hypomethylated gene (*Cotton_A_00760*) in diploid (*Ga*), *A2D5*, and five wild allotetraploids (*wGh*, *wGb*, *Gt*, *Gm*, and *Gd*). *RPKM* reads per kilobase per million. **e** CG methylation patterns of DMRs between *G. arboreum* (Ga) and G*. raimondii* (Gr) (mCG_A-D_ > 0.6) in Ga (*A*) and Gr (*D*) and in the wild allotetraploid *G. hirsutum* consisting of A subgenome (*As*) and D subgenome (*Ds*). Each row indicates one DMR between G*. raimondii* and *G. arboreum. Red* and *black* brackets indicate conserved DMRs (cDMRs, mCG_As-Ds_ > 0.6) and homoeologous DNA methylation changes (hDMCs, mCG_As-Ds_ < –0.6), respectively. **f** Fraction of the homoeologs with hDMCs and the homoeologs with cDMRs, respectively, showed expression bias

To test the effects of DNA methylation on polyploid diversification, we identified conserved sequences (2 kb or longer) between diploid and allotetraploid species and analyzed DMRs between them (see “Methods”). We found 100,246 CG, 109,424 CHG, and 252,042 CHH DMRs between allotetraploid and diploid species, ~30% of which were common to all five allotetraploid cottons (Additional file 2: Figure S4; Additional file 4: Table S3). The common DMRs in allotetraploid cottons except for CHH-hyper DMRs were enriched in genic and intergenic regions, but largely excluded from TEs (Fig. 4a).

For genes, CG-hyper DMRs were significantly enriched in the gene body, while CG-hypo DMRs were enriched in intergenic regions (Fig. 4a). In genic regions, A and D homoeologs of allotetraploid cottons showed higher CG methylation levels in the gene body (*P* < 1e-^100^, Wilcoxon signed-rank test) but lower CG methylation levels in the promoters (*P* < 1e-^100^, Wilcoxon signed-rank test) than diploid species (Fig. 4b). Among diploid species, CG methylation levels of the gene body were lower in A-genome *G. arboreum* than in D-genome *G. raimondii.* However, in allotetraploid cotton, CG methylation levels were higher in the A than in the D homoeologs (Fig. 4b), indicating that more CG methylation occurred in the A homoeologs of allotetraploid cotton. This is consistent with higher H3K4me3 levels in the D than in the A homoeologs in allotetraploid cotton [47], as intragenic DNA methylation antagonizes H3K4 trimethylation in plants [48, 49] and mammalian cells [50]. Using an absolute value of at least 0.6 in CG methylation changes between wild *G. hirsutum* and diploid species, we identified 501 hypermethylated and 137 hypomethylated genes in wild *G. hirsutum* compared with diploid species (Additional file 5: Table S4). CG methylation changes in the gene body significantly induce gene expression changes (Fig. 4c, d; Additional file 2: Figure S5), indicating a role for CG methylation in altering expression of these homoeologs in allotetraploids.

Polyploid formation often leads to intergenomic interactions including homoeologous exchanges [51], which are predicted to occur between methylated and unmethylated homoeologous loci. Among 5850 DMRs identified between the diploid species (A vs. D), 1165 DMRs (~20%) remained unchanged between A- and D-subgenome homoeologs (As vs. Ds) in the wild *G. hirsutum*, which designated conserved DMRs (cDMRs) (Fig. 4e). However, 339 DMRs (~6.8%) had opposite patterns between A and D homoeologs in the wild *G. hirsutum*, which designated homoeologous DNA methylation changes (hDMCs) (Fig. 4e). Interestingly, more homoeologs with hDMCs than the homoeologs with cDMRs displayed the expression bias (A/D ≠ As/Ds homoeologs) (Fig. 4f; Additional file 6: Table S5), indicating a role for hDMCs in homoeolog expression bias in the allotetraploids. We randomly selected three homoeolog pairs with hDMCs and expression bias, which were validated by bisulfite conversion and Sanger sequencing (Additional file 2: Figure S6), suggesting the reliability of these hDMCs.

During the evolution of allotetraploid cottons, some homoeolog pairs (A and D) lost one homoeolog [33], but the gene was present in both progenitor-like diploid species (*G. raimondii* and *G. arboreum*). To infer the role of DNA methylation in gene loss, we identified all homoeolog pairs with one copy lost in allotetraploid cottons but the corresponding orthologs present in both diploid species, and compared DNA methylation levels of these genes. Consistent with the previous finding of correlating DNA methylation with gene loss in paleopolyploid beans [30], the genes lost in the allotetraploids but present in diploid species showed significantly higher non-CG methylation levels in the gene body than their corresponding homoeologs that were retained in the allotetraploids (*P* < 1e-^20^, Wilcoxon signed-rank test) (Additional file 2: Figure S7). This suggests that non-CG methylation in the gene body could cause gene silencing and eventually gene loss.

### DNA methylation contributes to cotton domestication

**Fig. 5:**
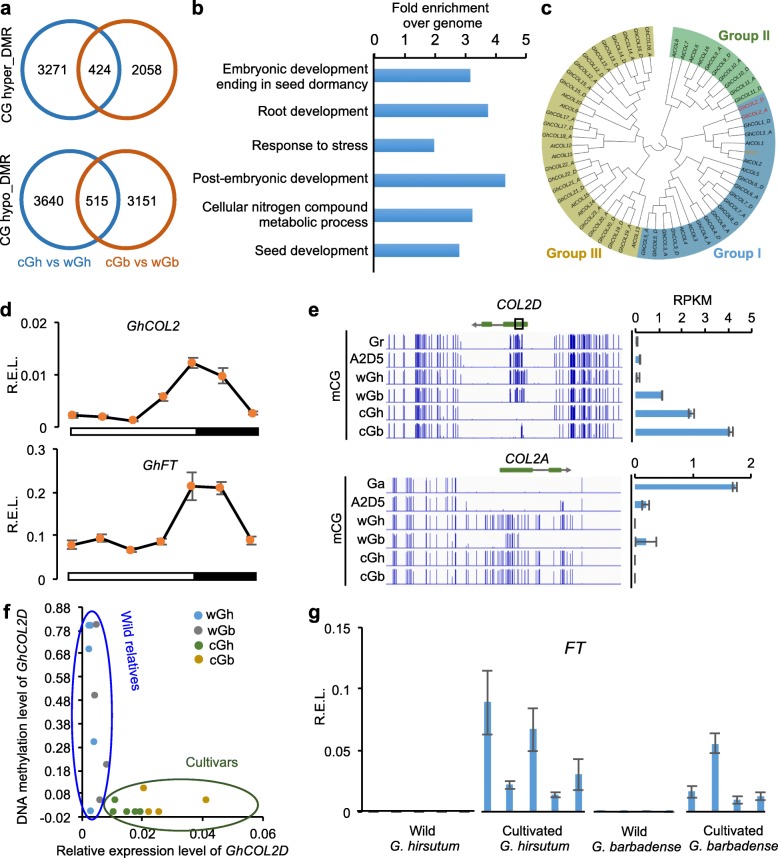
Demethylation of *COL2D* during cotton domestication. **a** Overlap of hyper DMRs (cGh-wGh or cGb-wGb, *upper panel*) or hypo DMRs (cGh-wGh or cGb-wGb, *lower panel*) in *G. hirsutum* and *G. barbadense*. **b** Gene Ontology (*GO*) enrichment of the genes overlapped with shared DMRs between wild and cultivated cottons. **c** Phylogenetic tree of *CONSTANS*-like genes in *Arabidopsis* and cotton. Gene IDs for each cotton *CONSTANS*-like gene are shown in Additional file 9: Table S8. **d** Relative expression levels (*R.E.L.* to *GhUBQ10*) of *GhCOL2* and *GhFT* in diurnal rhythms. *Black* and *white boxes*, respectively, indicate dark (16 h) and light (8 h) cycles. **e** During allotetraploid cotton domestication, reduced CG methylation levels correlated with increased expression levels in the *COL2D* locus, but *COL2A* was heavily methylated and silenced. Abbreviations are the same as in Fig. 1a. *RPKM* reads per kilobases per million. **f** DNA methylation levels in the *boxed region* of *COL2D* in **e** (D03: 32225460-32225724) (*y*-axis) and relative expression levels of *COL2D* in nine wild and nine cultivated cotton accessions. **g** R.E.L. (to *GhUBQ10*) of *FT* in five wild and five cultivated *G. hirsutum* and *G. barbadense* lines

Cultivated *G. hirsutum* and *G. barbadense* possessed 7850 and 6148 CG DMRs, respectively, relative to their wild relatives (Additional file 7: Table S6). However, most DMRs (>85%) were specific to *G. hirsutum* or *G. barbadense*, and only ~14% were common to both species (Fig. 5a), consistent with the notion that *G. hirsutum* and *G. barbadense* were independently domesticated [35]. The shared DMRs were associated with 519 genes, which could generate putative epialleles (Additional file 8: Table S7). Gene Ontology (GO) analysis showed that these genes were enriched in several important biological processes, including metabolic processes, stress response, and regulation of seed dormancy (Fig. 5b). For example, cotton homologs of the *Arabidopsis* genes encoding TARGET OF RAPAMYCIN (TOR) and its binding partner RAPTOR were both demethylated in cultivated cottons but not in wild cottons (Additional file 8: Table S7). TOR, encoding a kinase, is a master regulator to adjust cell growth and development in response to nutritional signaling in plants and animals [52]. In *Arabidopsis*, root and shoot growth, seed yield, and stress tolerance are positively correlated with *TOR* expression levels [53, 54]. Disruption of *TOR* or *RAPTOR* leads to abnormal vegetative growth and seed abortion. In contrast, plants with moderate increase of *TOR* expression show enhanced root and shoot growth, greater resistance to osmotic stress, and higher seed production. These epialleles identified in tetraploid cottons could contribute to morphological and physiological changes, including fiber length, reduction in seed dormancy, and photoperiod sensitivity [28], during domestication of *G. hirsutum* (Upland) and *G. barbadense* (Pima) cotton.

Among the putative epialleles, we selected a cotton *CONSTANS-LIKE2D* homoeolog (*COL2D*) and an *Arabidopsis CONSTANS* (*CO*) homolog [55] for functional analysis. In *Arabidopsis*, *CO* and *CO-LIKE* (*COL*) genes control photoperiodic flowering through induction of *FLOWERING LOCUS T* (*FT*), and CO regulates *FT* expression through diurnal rhythms [55–57]. Phylogenetically, eight *G. hirsutum COLs* (*GhCOLs*) (1–8, group I *COL*) were in the same clade with *Arabidopsis CO* (Fig. 5c; Additional file 9: Table S8). Among *GhCOLs*, only *GhCOL2* exhibited similar expression rhythms with *GhFT*, indicating that *GhCOL2* is a major regulator of *GhFT* (Fig. 5d; Additional file 2: Figure S8).

Loss of photoperiod sensitivity is a major “domestication syndrome” trait [35] of Upland or American cotton (*G. hirsutum* L.) and Pima or Egyptian cotton (*G. barbadense* L.) that accounts for >95% and ~5%, respectively, of these annual cotton crops worldwide [31]. We predicted that DMRs of *COL2D* could lead to the loss of photoperiod sensitivity in domesticated cottons and promote global cotton production. Indeed, lower CG methylation levels of *COL2D* were associated with its higher expression levels in cultivated and photoperiod-insensitive *G. hirsutum* and *G. barbadense* than in their wild relatives (photoperiod sensitive) (Fig. 5e). To confirm the relationship between loss of DNA methylation and expression of *COL2D*, we treated the wild *G. hirsutum* (TX2095) seedlings using 5-aza-2′-deoxycytidine (5-aza-dC), a chemical inhibitor for DNA methylation [58]. Consistent with the hypothesis that demethylation of *COL2D* leads to increased levels of expression, the 5-aza-dC treatment reduced DNA methylation and increased expression levels of *COL2D* (Additional file 2: Figure S9). Although we cannot rule out possible toxic and side effects of 5-aza-dC, both genome-wide data and 5-aza-dC treatment results indicate that DNA methylation loss in the *COL2D* could promote its expression in domesticated cottons. Notably, *COL2D* was heavily methylated and silenced in *G. raimondii* that is photoperiod sensitive, while *COL2A* was hypomethylated and highly expressed in cultivated *G. arboreum* (Fig. 5e), which is photoperiod insensitive [59]. Interestingly, the *COL2A* homoeolog was hypermethylated and repressed in cultivated Upland and Pima cottons, while the *COL2D* homoeolog was highly expressed (Fig. 5e), suggesting that *COL2A* in the allotetraploid cottons was either silenced after polyploid formation or originated from a progenitor that was different from the extant *G. arboreum* species. The high-level expression of *COL2D* is likely associated with positive selection of *COL2D* but not with *COL2A* during domestication of Upland and Pima cottons [60].

Consistent with the role of this epiallele in allotetraploid cotton domestication, locus-specific DNA methylation loss and expression increase of *COL2D* were associated with nine cultivated accessions but not with nine wild accessions of *G. hirsutum* and *G. barbadense* which were randomly selected for this test (Fig. 5f). As a result, cotton *FT* was expressed at higher levels in the cultivated accessions than in their wild relatives (Fig. 5g).

**Fig. 6:**
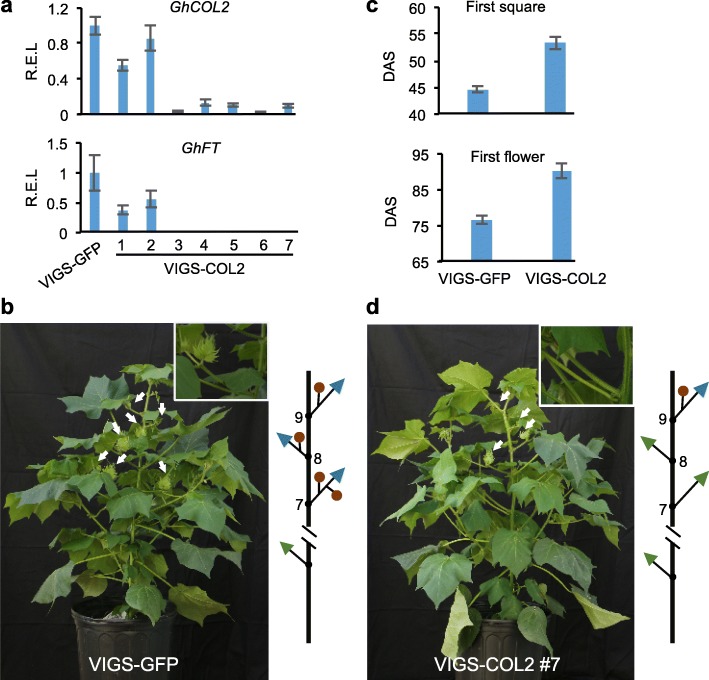
Repression of *COL2* delayed flowering of cultivated cotton (TM-1). **a** Relative expression levels (*R.E.L.*) of *GhCOL2* (*upper panel*) and *GhFT* (*lower panel*) in seven lines of virus-induced gene silencing (*VIGS*) of *COL2D*. R.E.L. were normalized to those of the VIGS-GFP control. **b** A control plant of VIGS-GFP lines and a diagram to indicate the branch location (node 7) of the first square (immature flower) that appeared. **c** Days after sowing (*DAS*) when the first square (*upper panel*) and first flower (*lower panel*) appeared in the control (VIGS-*GFP*) and VIGS-*COL2* lines (*n* = 5), respectively. **d** Plants of *COL2-*VIGS lines showed delayed flowering (at node 9), as shown in the diagram. *Insets*: enlarged views of node 7; *white arrows* indicate square locations. *Green* and *blue arrowheads* indicate monopodial and sympodial branches, respectively; *brown balls* indicate cotton squares. Nodes (7–9) with squares are indicated

If *COL2* regulates photoperiodic flowering in cotton, reducing *COL2* expression in Upland cotton (accession TM-1) would delay flowering. This should mimic the methylation effect on a specific gene, which cannot be easily tested. Using virus-induced gene silencing (VIGS) [61], *COL2* was specifically down-regulated (Fig. 6a), while expression of other *COLs* remained unchanged relative to the control plants that expressed VIGS of GFP (VIGS-GFP) (Additional file 2: Figure S10). Expression of the downstream gene *GhFT* was also inhibited in seven independently derived VIGS lines of *COL2* (VIGS-*COL2* #1-7), mimicking the repression pattern of *COL2D* (Fig. 6a). In addition, knocking down expression of other *COLs* such as *GhCOL3* and *GhCOL6* did not affect *GhFT* expression (Additional file 2: Figure S11), further validating this specific role of *COL2* in regulating *GhFT* expression in cotton. Down-regulating *COL2* and *GhFT* expression has delayed flowering time in five VIGS-GhCOL2 lines (#3–7) analyzed (Fig. 6b, c). In the control plants, the first square appeared at ~44 days after sowing (DAS) on node 7 of the main stem in the long-day condition (Fig. 6b, c), while the first squares emerged by ~53 DAS on node 9 in the VIGS-*COL2* lines. Consequently, VIGS-*COL2* also flowered later than the control plants (Fig. 6d). As wild cottons normally do not flower in the long-day condition [60], delayed flowering by knocking down *COL2* expression indicates that other genes could also mediate photoperiodic flowering in cotton. This notion is supported by several quantitative trait loci (QTLs) that are associated with the loss of photoperiod sensitivity during cotton domestication [62, 63].

## Discussion and conclusions

DNA methylation affects many biological processes including disease-associated syndromes in humans [64]. Natural variation of epialleles has emerged to play a role in plant evolution [65], morphological diversity in plants [11], and selection and breeding of agronomic traits in crops [12, 13]. The evolutionary history of cotton species consists of four intervened phases: genome variation among diploid species including A- and D-genome progenitors, interspecific hybridization and allotetraploid formation, natural selection and adaptation, and artificial selection and domestication (Fig. 1a). In this study, we compared DNA methylation changes between Gr and Ga, A2D5 hybrid and Gr/Ga, wild tetraploid cottons (wGh, wGb, Gt, Gd, and Gm) and diploid cottons (Gr and Ga), and cultivated cottons (Gh and Gb) and wild tetraploid cottons (wGh and wGb) and examined the role of DNA methylation in four evolutionary phases. We found that the rate of DNA methylation changes exceeds that of neutral sequence substitutions during cotton evolution and domestication (Fig. 1d, e). The methylated genes remain methylated with a high rate of DNA methylation changes in the coding sequences, whereas unmethylated genes stay unmethylated with little or no change in DNA methylation. This probably occurs because, for unmethylated genes, DNA methylation is dispensable for expression regulation, and *cis*-acting elements and/or *trans*-acting factors may regulate expression of these genes. For methylated genes, DNA methylation could change and alter gene expression in response to internal (genetic perturbation) and external (environmental variation) signals during evolution and adaptation. This suggests that the DNA methylation status of genes in ancestral progenitor species could be selected and maintained during evolution.

The content and organization of repeats and transposons in the genome are correlated with DNA methylation patterns, which could affect transcription activity of neighboring genes [66, 67]. Among diploid species, *G. raimondii* has more TEs and higher DNA methylation levels in genic regions than *G. arboreum*, although *G. arboreum* has more TEs in the whole genome [33, 41]. The asymmetrical expansion of TEs and DNA methylation is probably a rule for the evolution of ancestral cotton genomes. Interestingly, although TEs are associated with higher DNA methylation levels than other regions, DNA methylation changes between diploid and allotetraploid cottons were largely excluded from TEs in conserved sequences between diploid and allotetraploid cottons (Fig. 4a), indicating that DNA methylation in TEs is relatively stable during evolution. To exclude a potential effect of sequence variation on DNA methylation analysis, we used the conserved sequences to examine DNA methylation changes between diploid and allotetraploid cottons. DNA methylation changes in non-conserved sequences should be examined in the future by longer reads, such as single-molecule real-time (SMRT) sequencing [68].

Hybridization and polyploidy play key roles in plant speciation and diversification [4, 8]. Hybridization induces genome-wide DNA methylation changes [27, 44]; however, it is unclear whether these changes could be maintained in the long-term evolution of allopolyploids. Remarkably, we observed that the majority of DNA methylation changes induced in the interspecific hybrids are maintained in one or more allotetraploid cottons, some of which are shared between wild and domesticated cottons, suggesting that hybridization-induced DNA methylation changes are transmittable during polyploid speciation. Furthermore, we uncovered homoeologous DNA methylation changes, which correlate with homoeolog-expression bias, providing an epigenetic basis for gene expression and evolutionary novelty in the polyploids [69]. This is reminiscent of homoeologous sequence or chromosomal exchanges between subgenomes that occur in some polyploids like *Brassica* [51] or *Tragopogan* [70]. Homoeologous DNA methylation changes (hDMCs) in allotetraploid cottons could occur from genomic exchanges, although this type of intergenomic change is rather rare in polyploid cotton [71] or *Arabidopsis* [72, 73]. Alternatively, small interfering RNAs (siRNAs) produced from either or both homoeologs could target a different homoeolog to induce de novo methylation via RdDM [74, 75] which is maintained during evolution.

Consistent with the stable maintenance of DNA methylation, many candidate epialleles could contribute to morphological and physiological changes during cotton evolution and domestication, providing a valuable resource for epigenetic engineering and breeding better-yielding crops. This example of the domestication-induced epiallele *COL2D* that contributes to the loss of photoperiod sensitivity by demethylation in cultivated cottons provides novel insights into the role of DNA methylation in domestication of cotton and many other polyploid crops. Functional analysis of these polyploidy-induced epialleles should help develop biotechnological tools for manipulating DNA methylation and epialleles through breeding, selection, and ultimate improvement of plants and animals.

## Methods

### Plant materials

*G. raimondii* (Gr, D5-3), *G. arboreum* (Ga, A2), interspecific hybrid (A2D5) between *G. arboreum* and *G. raimondii*, wild *G. hirsutum* (wGh, TX2095), wild *G. barbadense* (wGb, Gb-706), *G. tomentosum* (Gt, AD3-30), *G. mustelinum* (Gm, AD4-11), *G. darwinii* (Gd, AD5-31), cultivated *G. hirsutum* (cGh, TM-1), and cultivated *G. barbadense* (cGd, Pima-S6) were grown in the greenhouses at College Station and Austin, Texas. Leaves of each genotype were harvested with three biological replications for MethylC-seq and RNA-seq libraries. DNA methylation and expression levels of *GhCOL2_D* were further analyzed in five wild *G. hirsutum* accessions (TX701, TX1039, TX2092, TX2095, and TX2096), five cultivated *G. hirsutum* (TM-1, SA-308, SA-508, SA-528, and SA-1475), four wild *G. barbadense* (Gb-472, Gb-470, Gb-617, Gb-716), and four cultivated *G. barbadense* (Pima S2, Pima S6, Phytogen 800, 3-79), which were grown in the greenhouse under the light/dark (L/D) cycle of 16 h/L at 24 °C and 8 h/D at 20 °C. To exclude effects of development stage and circadian rhythm on gene expression change, the first true leaves at 16 days after sowing were harvested at ZT15 (zeitgeber time, ZT0 = dawn, 6 am) for DNA and RNA extraction.

### mRNA-seq library construction

After DNase treatment, total RNA (~1 μg) was subjected to construct strand-specific mRNA-seq libraries with two biological replications using NEBNext® Ultra™ Directional RNA Library Prep Kit (New England Bioloabs (NEB), Ipswich, MA, USA) according to the manufacturer’s instructions. For each genotype, mRNA-seq libraries were constructed with two biological replicates and were paired-end sequenced for 126 cycles.

### MethylC-seq library construction

Total genomic DNA (~5 μg) was fragmented to 100–1000 bp using Bioruptor (Diagenode, Denville, NJ, USA). End repair (NEBNext® End Repair Module) was performed on the DNA fragments followed by adding an ”A” base to the 3′ end (NEBNext® dA-Tailing Module), and the resulting DNA fragments were ligated to the methylated DNA adapter (NEXTflex™ DNA Barcodes, Bioo Scientific, Austin, TX, USA). The adapter-ligated DNA of 200–400 bp was purified using AMPure beads (Beckman Coulter, Brea, CA, USA), followed by sodium bisulfite conversion using the MethylCode™ Bisulfite Conversion Kit (Life Technologies, Foster City, CA, USA). The bisulfite-converted DNA was amplified by 12 cycles of PCR using LongAmp® Taq DNA Polymerase (NEB) and subject to purification using AMPure beads (Beckman Coulter). For each genotype, MethylC-seq libraries were constructed with two biological replicates and paired-end sequenced for 126 cycles.

### qRT-PCR

After DNase treatment, total RNA (2 μg) was used to produce cDNA with the Omniscript RT Kit (Qiagen, Valencia, CA, USA). The cDNA was used as the template for qRT-PCR using FastStart Universal SYBR Green Master (Roche, Indianapolis, IN, USA). The reaction was run on the LightCycler® 96 System (Roche, Pleasanton, CA, USA). The relative expression level of a gene was quantified using the expression value of cotton *GhUBQ10* as an internal control using the primers (Additional file 10: Table S9).

### Analysis of mRNA-seq data

mRNA-seq reads of *G. raimondii* and *G. arboreum* were mapped to genome sequences of *G. raimondii* and *G. arboreum,* respectively [40, 41]. mRNA-seq reads of other species were mapped to combined genome sequences of *G. raimondii* and *G. arboreum*. TopHat software with options (--library-type fr-firststrand --b2-score-min L, 0, -0.6) was used for read mapping [76]. Uniquely mapped reads were extracted and analyzed by Cufflinks to determine transcript values [77]. The differentially expressed genes (DEGs) were identified using both the fold-change (>twofold) and analysis of variance (ANOVA) tests (*P* < 0.01) with two biological replicates.

### Mapping of MethylC-seq reads with two biological replicates

MethylC-seq reads of *G. raimondii* and *G. arboreum* were mapped to genome sequences of *G. raimondii* and *G. arboreum,* respectively [40, 41]. MethylC-seq reads of the A2D5 hybrid were mapped to the combined genome sequences of *G. raimondii* and *G. arboreum*. MethylC-seq reads of all allopolyploid cottons were mapped to the genome sequence of *G. hirsutum* (TM-1) [33]. Bismark with parameters (--score_min L,0,-0.2 -X 1000 --no-mixed --no-discordant) was used for read mapping [78]. Only reads mapped to the unique sites were retained and used for further analysis. The reads mapped to the same site were collapsed into a single consensus read to reduce clonal bias.

### Conserved cytosines between diploid and allotetraploid cottons

To exclude the bias, we selected the conserved regions (2 kb or longer) by aligning *G. hirsutum* (tetraploid) genome sequences with *G. arboreum* and *G. raimondii* genome sequences (Additional file 2: Figure S1). Specifically, genome sequences of *G. raimondii* and *G. arboreum* were aligned to the genome sequence of *G. hirsutum* (TM-1) using LAST (version 545) software (*lastal –q3 –e35 –m50*) [79]. The unique best alignments with score >500 were extracted (*last-split –m1 –s200*). The 1-to-1 alignments were generated by swapping the sequences (*maf-swap*) and subjected to extracting best alignments again (*last-split –m1 –s200*) until the alignments reached a score over 2000, and the conserved cytosines between diploid and allopolyploid cottons were extracted using Python scripts. Thus, these conserved regions are present in both diploid and tetraploid cottons. DMRs identified between allotetraploids and diploid species were present in all allotetraploid and diploid cottons.

### Conserved cytosines among allotetraploid cottons

To avoid the base bias among different species, only the conserved cytosines were used for the analysis. Although most cytosines were changed to uracil in bisulfite conversion, guanine in MethylC-seq reads could be used to confirm cytosine in the complementary strand (Additional file 2: Figure S1c). After mapping MethylC-seq reads of wild *G. hirsutum*, *G. barbadense*, *G. tomentosum*, *G. mustelinum*, and *G. darwinii* to the reference genome of cultivated *G. hirsutum* (TM1) [33], guanines (G) in uniquely mapped MethylC-seq reads were used to confirm cytosines (C) in the complementary strand. All conserved cytosines were called when the same bases were covered by at least three reads and used for further analysis.

### Phylogenetic tree

Methylation levels of the cytosines conserved in all species were obtained in each species. We calculated the distance between every two species using Euclidean distances. For two species *P* and *Q*, *P* = (p_*1*_, p_*2*_, …, p_*n*_) and *Q* = (q_*1*_, q_*2*_, …, q_*n*_) in *n* CG sites, p_*n*_ is the methylation level in the *n*th CG site in the *P* species; q_*n*_ is the methylation level in the *n*th CG site in the *Q* species. The general Euclidean distance between *P* and *Q* was calculated as ((p_*1*_-q_*1*_)^^2^ + (p_*2*_-q_*2*_)^^2^ + … + (p_*n*_-q_*n*_)^2)^^0.5^. The Euclidean distances were used to construct the distance matrix for all species. Phylogenetic trees were constructed based on the distance matrix using the ‘ape’ R package with Neighbor-Joining algorithm and 1000 bootstraps [80].

### Differentially methylated cytosines (DmCs)

Only cytosines covered by at least three reads in both species in comparison were considered for testing. The methylation level of a cytosine site was calculated as C/(C + T) [81]. C indicates the number of reads with cytosine for this site. T indicates the number of reads with thymine for this site. A cytosine was considered as a DmC between two species if it showed an absolute change in methylation level of at least 0.5 and *P* < 0.01 by a one-way ANOVA test between species using two biological replicates.

### Differentially methylated regions (DMRs)

DMRs were identified using 100-bp sliding windows. The mean methylation level was calculated for each window [81]. For CG and CHG DMRs, windows containing at least four cytosines in the CG or CHG context covered by at least three reads were selected for identifying DMRs. For CHH DMRs, windows containing at least 16 cytosines in the CHH context covered by at least three reads were selected. DMRs between two species in comparison were determined using an ANOVA test with two biological replications (*P* < 0.05) and cut-off values of methylation levels (0.5 for CG and CHG DMRs; 0.2 for CHH DMRs).

### Identification of orthologous genes

Orthologous genes were obtained from a published paper [33]. Orthologous genes with more than 40% cytosines in the gene body covered by at least three reads were selected to calculate *K*s and DmCG ratios between methylated and unmethylated orthologous genes.

### Treatment of cotton seedling using 5-aza-2′-deoxycytidine (5-aza-dC)

In a beaker, cotton seeds (TX2095) were placed on sterile gauze and soaked in water with or without 5-aza-dC (18 mg/L). The beaker was capped with plastic wrap and placed in a climate incubator under a light/dark (L/D) cycle of 16 h/L at 30 °C and 8 h/D at 24 °C. At 3 days after sowing (DAS), the old gauze was replaced with new gauze soaked in water with or without 5-aza-dC. From 6 DAS, we replaced the old gauze with new gauze soaked in 1/2 Murashige and Skoog medium with or without 5-aza-dC (18 mg/L) every 3 days. The first true leaves at 16 DAS were harvested at ZT15 with five biological replicates for DNA and RNA extraction.

### Bisulfite sequencing of *COL2D* fragment

Approximately 500 ng of genomic DNA was used for bisulfite conversion using the MethylCode™ Bisulfite Conversion Kit (Life Technologies). Bisulfite-treated DNA was then amplified by ZymoTaq DNA polymerase (Zymo Research, Irvine, CA, USA) and primers (5′-TTATTTGTAGTGTTGATGTAGTATTATTTTG-3′ and 5′-TTTCCAAACTCAAACAATAACCAAAAATCCATTTC-3′) targeting the *COL2D* fragment (D03: 32225460-32225724). The purified amplicons were cloned into a pGEM-T vector (Promega, Madison, WI, USA). For each plant genotype, an average of 15 clones was randomly chosen for sequencing in two biological replicates.

### Virus-induced gene silencing (VIGS)

*G. hirsutum* acc. TM-1 grew in a greenhouse under the light/dark (L/D) cycle of 16 h/L at 24 °C and 8 h/D at 20 °C. Fragments of *GhCOL2*, *GhCOL3*, and *GhCOL6* cDNA were amplified by PCR using primers (Additional file 10: Table S9) and subsequently cloned into the pYL156 (pTRV-RNA2) vector as pYL156-GhCOL2, pYL156-GhCOL3, and pYL156-GhCOL6, respectively. The plasmid pYL156-GFP was used as a control [61]. The plasmids pTRV-RNA1, pYL156-GFP, pYL156-GhCOL2, pYL156-GhCOL3, and pYL156-GhCOL6 were transformed into *Agrobacterium tumefaciens* strain GV3101 by electroporation. The *Agrobacterium tumefaciens* strain GV3101 was incubated overnight in Luria-Bertani medium containing 10 mM MES and 20 uM acetosyringone and finally resuspended in infiltration buffer (10 mM MES, 0.2 mM acetosyringone, 10 mM MgCl_2_) to a final concentration of OD600 = 1.0. Cell suspensions were incubated at room temperature for 3 h. Equal amounts of different bacterial suspensions (pTRV-RNA1 with pYL156-GFP, pYL156-GhCOL2, pYL156-GhCOL3, or pYL156-GhCOL6) were infiltrated into the fully expanded cotyledons of the 10-day-old cottons with a needleless syringe.

## Additional files

Additional file 1: Table S1. Summary of MethylC-seq reads. (XLSX 10 kb)

Additional file 2: Supplemental **Figures S1**–**S11**. (PDF 438 kb)

Additional file 3: Table S2. DMRs between A2D5 hybrid and the parents (Gr/Ga). (XLSX 2969 kb)

Additional file 4: Table S3. DMRs between polyploid and diploid (Gr/Ga) cottons. (XLSX 19969 kb)

Additional file 5: Table S4. Differentially methylated genes between wGh and diploid cottons (Gr/Ga). (XLSX 47 kb)

Additional file 6: Table S5. Homoeologs with hDMCs showing expression bias. (XLSX 17 kb)

Additional file 7: Table S6. DMRs between cultivated and wild cottons. (XLSX 579 kb)

Additional file 8: Table S7. Genes overlapped with shared DMRs between wild and cultivated cotton. (XLSX 54 kb)

Additional file 9: Table S8. Gene ID for *GhCOLs*. (XLSX 9 kb)

Additional file 10: Table S9. Primers for qRT-PCR and VIGS. (XLSX 9 kb)
