# Supplementary material for: Epigenomic and functional analyses reveal roles of epialleles in the loss of photoperiod sensitivity during domestication of allotetraploid cottons
Source: Genome Biol. 2017 May 31;18:99. doi: 10.1186/s13059-017-1229-8 (PMC5450403; doi:10.1186/s13059-017-1229-8)
Supplement: Supplementary file 2 — Supplemental Figures S1–S11. (PDF 438 kb) [file 13059_2017_1229_MOESM2_ESM.pdf]

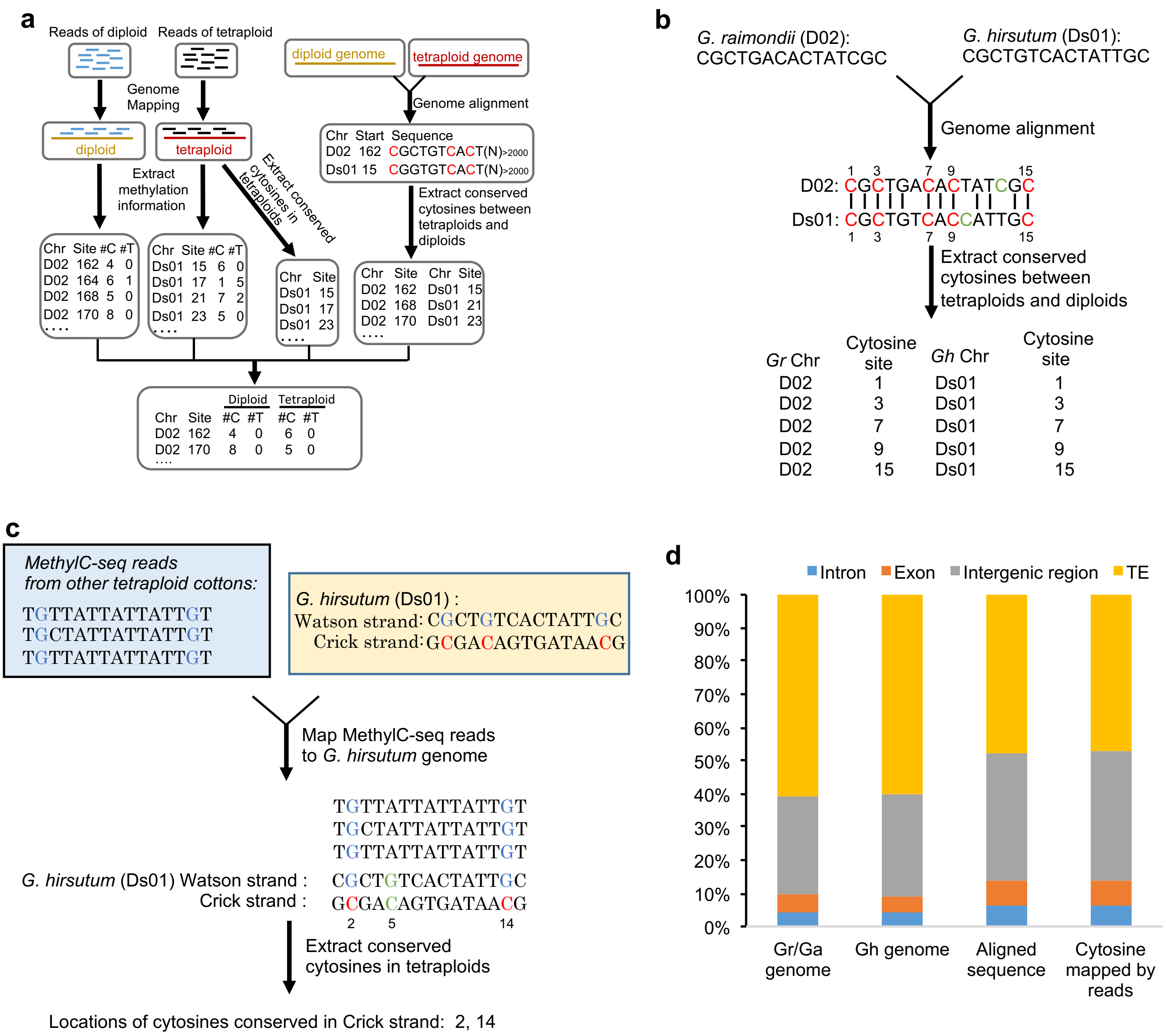

Figure S1: Identification of cytosines conserved in all species. (a) Pipeline of MethylC-seq read mapping and extraction of conserved cytosines. #C: number of mapped cytosines for each cytosine position. #T: number of mapped thymines for each cytosine position. **(b)** Schematic diagram of extracting conserved cytosines between diploid and tetraploid cottons. Red cytosine indicates conserved cytosine between diploid and tetraploid cottons. Green cytosine indicates nucleotide variation between diploid and tetraploid cottons. **(c)** Schematic diagram shows how to identify conserved cytonsines among tetraploids using guanine base in MethylC-seq reads. Red cytosine indicates conserved cytosine among tetraploids. Green cytosine indicates nucleotide variation among tetraploids. **(d)** Distribution (%) of cytosines in *G. arboreum* (Ga)/*G. raimondii* (Gr), *G. hirsutum* (Gh), aligned genomes and cytosines mapped by MethylC-seq reads. Aligned genome: sequences conserved between diploid and allopolyploid cottons.

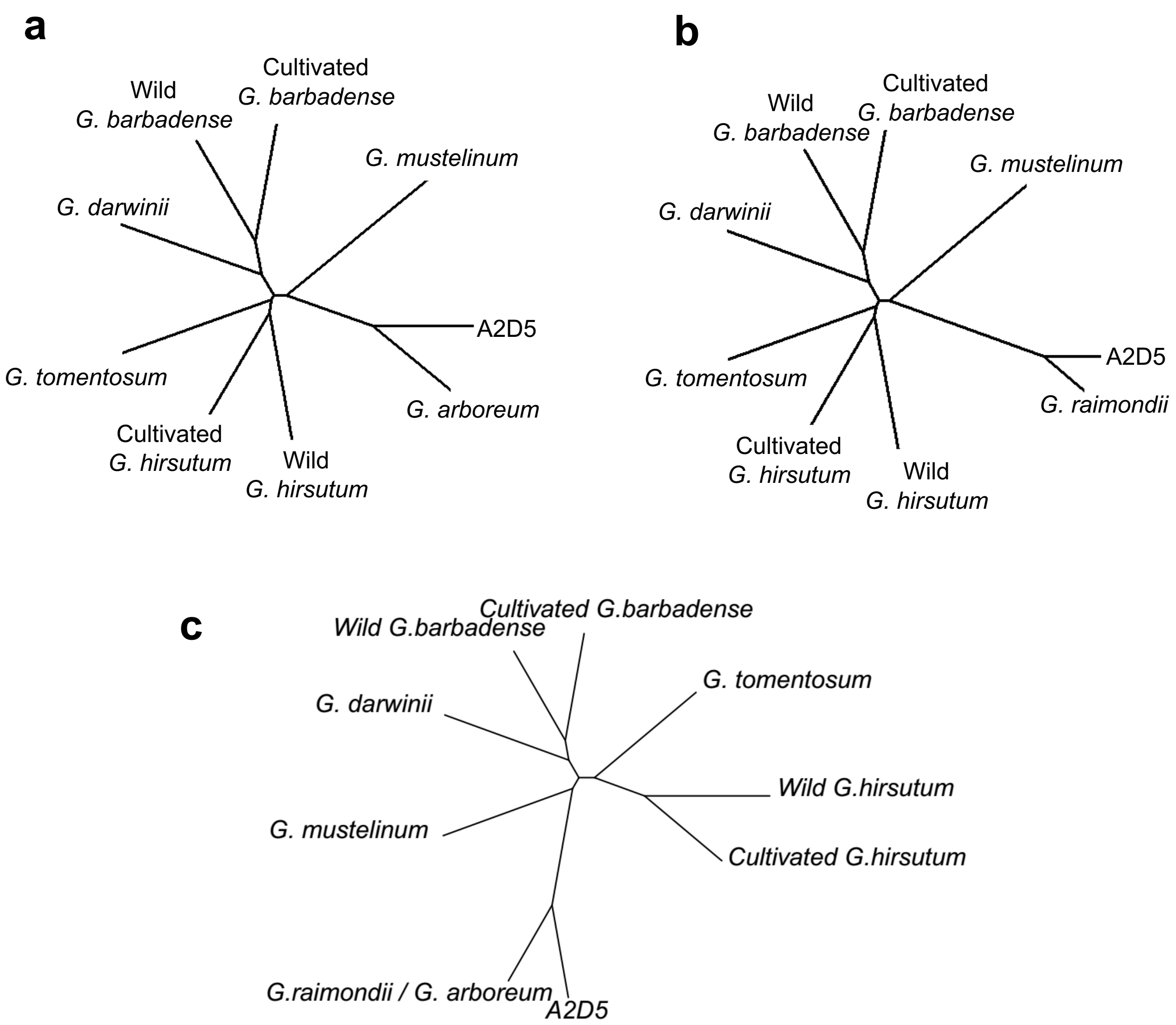

Figure S2: Phylogenetic trees based on DNA methylation. (a) Phylogenetic tree based on methylation divergence of A genome. **(b)** Phylogenetic tree based on methylation divergence of D genome. **(c)** Phylogenetic tree based on non-CG methylation divergence.

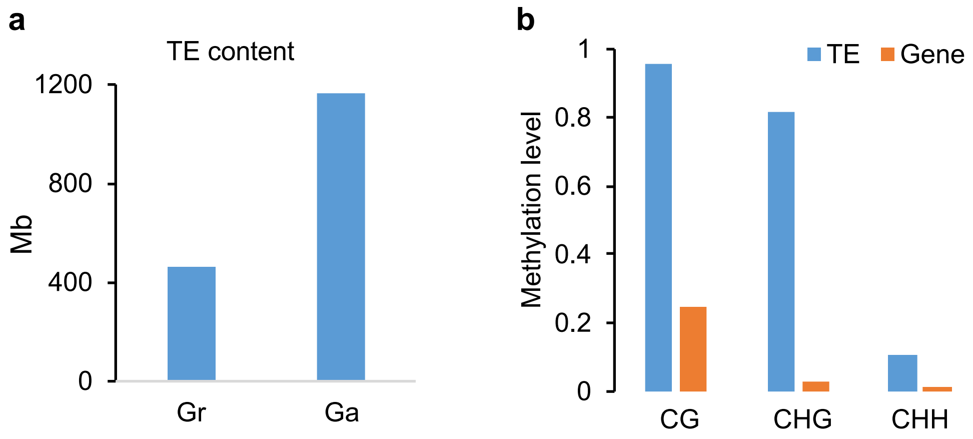

Figure S3: DNA methylation in TEs. (a) TE content in the genomes of *G. arboreum* (Ga) and *G. raimondii* (Gr). **(b)** DNA methylation levels of genes and TEs in *G. arboreum*.

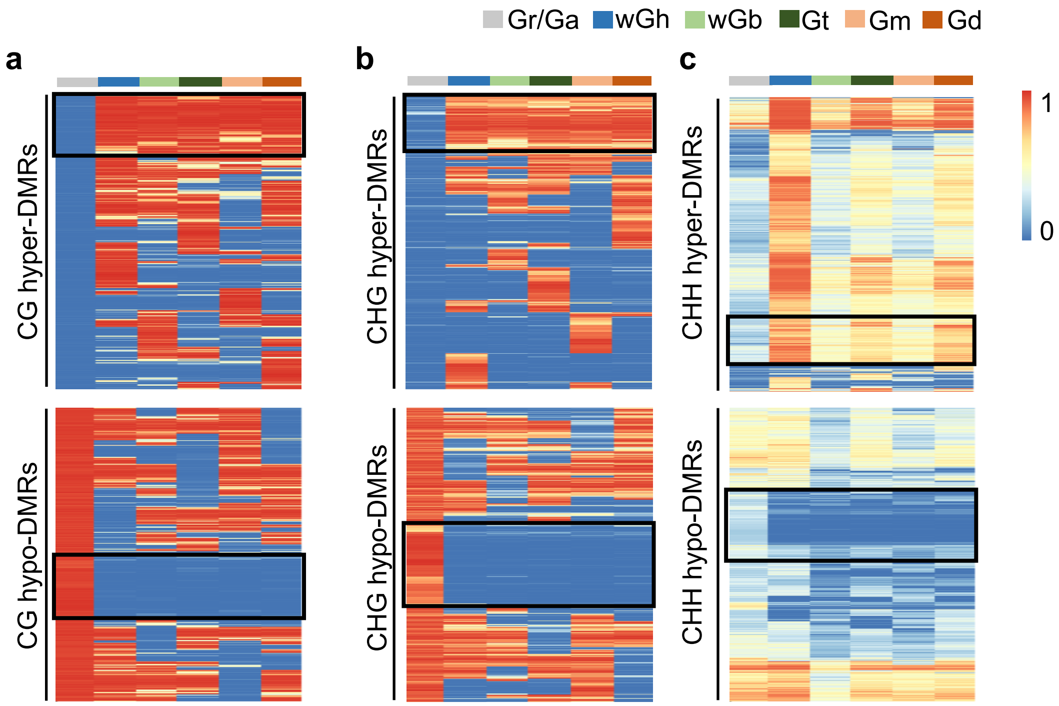

Figure S4: DMRs between allopolyploid cottons and diploid progenitors. Methylation levels of CG (**a**), CHG (**b**) and CHH (**c**) DMRs between allopolyploid cottons (wild *G. hirsutum*, wGh, wild *G. barbadense*, wGb,*G. tomentosum*, Gt, *G. mustelinum*, Gm, *G. darwinii*, Gd) and diploid progenitors (*G. arboreum,* Ga and *G. raimondii,* Gr). Black rectangles indicated DMRs shared by all allopolyploid cottons. Color bar indicate z-scores.

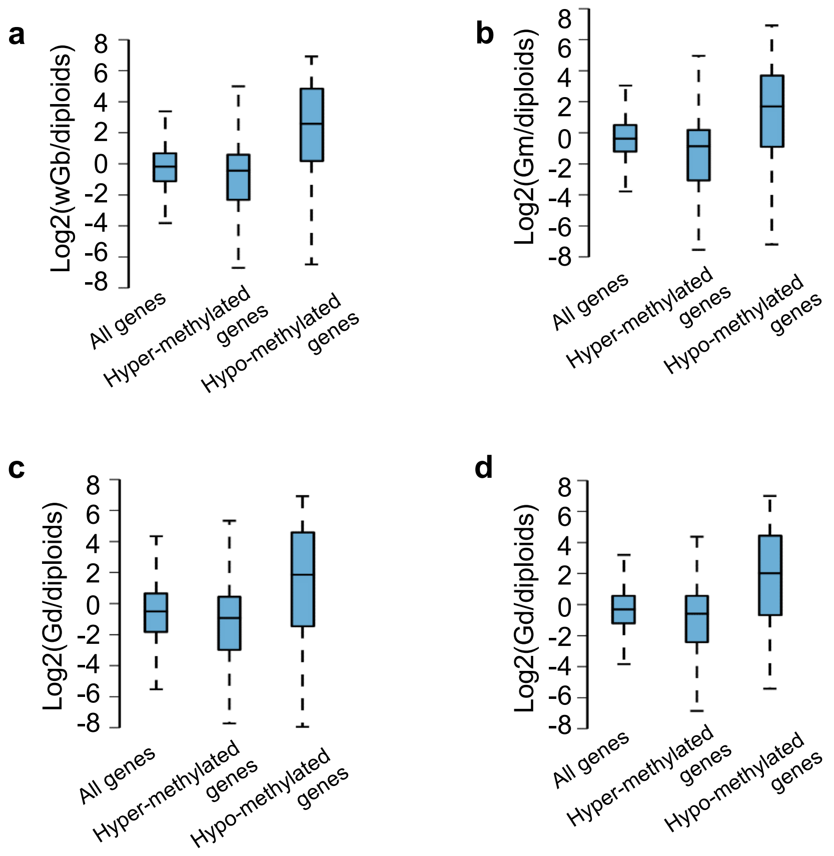

Figure S5: The relationship of gene expression changes and DNA methylation changes in wild *G. barbadense* (**a**), *G. mustelinum* (**b**), *G. tomentosum* (**c**), *G. darwinii* (**d**) compared with wild diploid species.

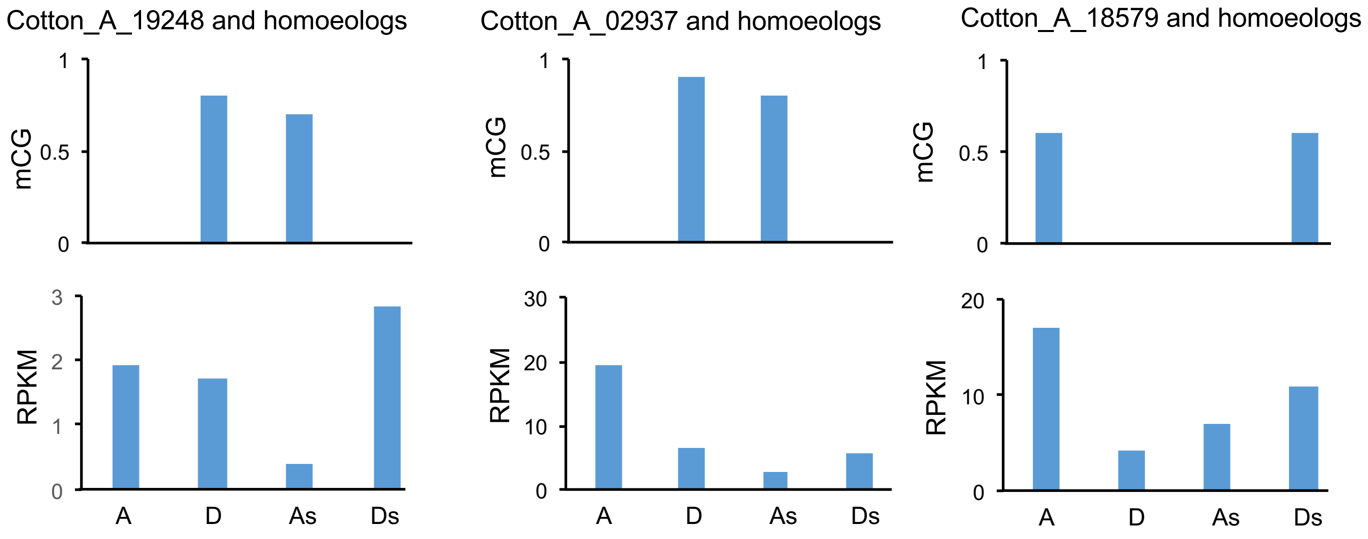

Figure S6: Confirm CG methylation levels for randomly selected genes with hDMCs using bisulfite sanger sequencing. The regions with different CG methylation levels for homoeologs were listed in Table S5 and the CG methylation levels of these regions were calculated by bisulfite sanger sequencing. A: *G. arboreum*. D: *G. raimondii*. As: A subgenome of wild *G. hirsutum*. Ds: D subgenome of wild *G. hirsutum*. RPKM: reads per kilobases per million. Each homoeolog ID was listed in Table S5. Primers for bisulfite sanger sequencing were listed in Table S9.

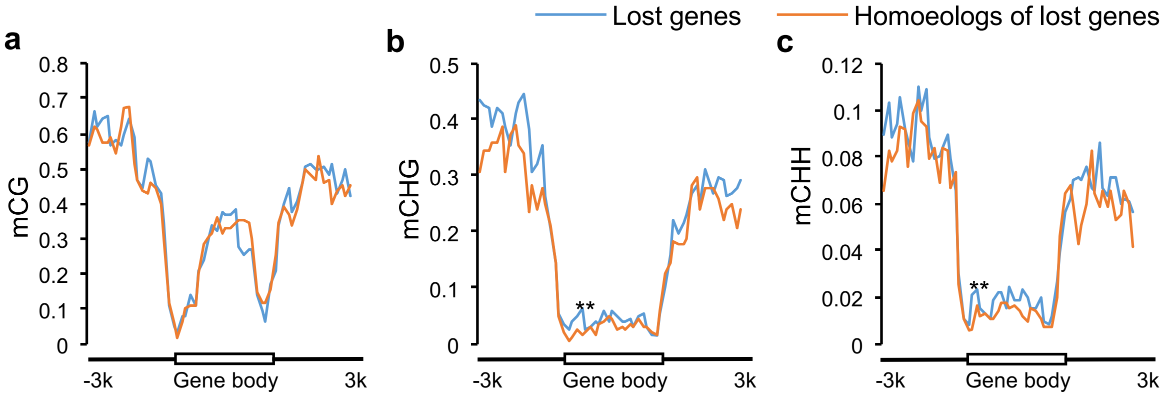

Figure S7: DNA methylation differences between the lost genes and their homoeologs that are retained during polyploidization in cotton. Lost genes showed similar CG (**a**) but significant higher CHG (**b**) and CHH methylation (**c**) in the gene body compared with their homoeologs.

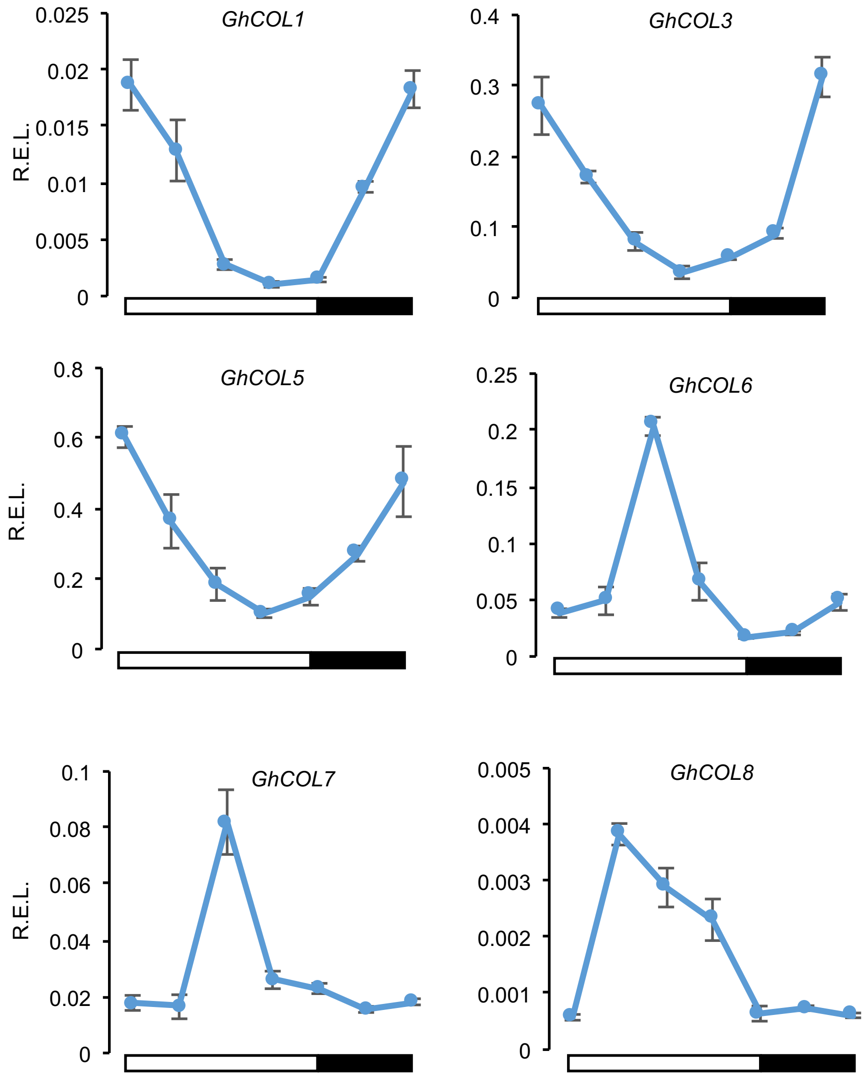

Figure S8: Diurnal expression patterns of *COL* genes. Relative expression levels (R.E.L.) of *GhCOL* genes (from *G. hirsutum* TM-1) in every 4 hours within a 24-hour diurnal cycle under the long day condition (16h light / 8h dark). Black and white boxes respectively indicate dark and light period of time. Expression levels of *GhCOL* genes and *GhFT* were normalized to *GhUBQ10.*

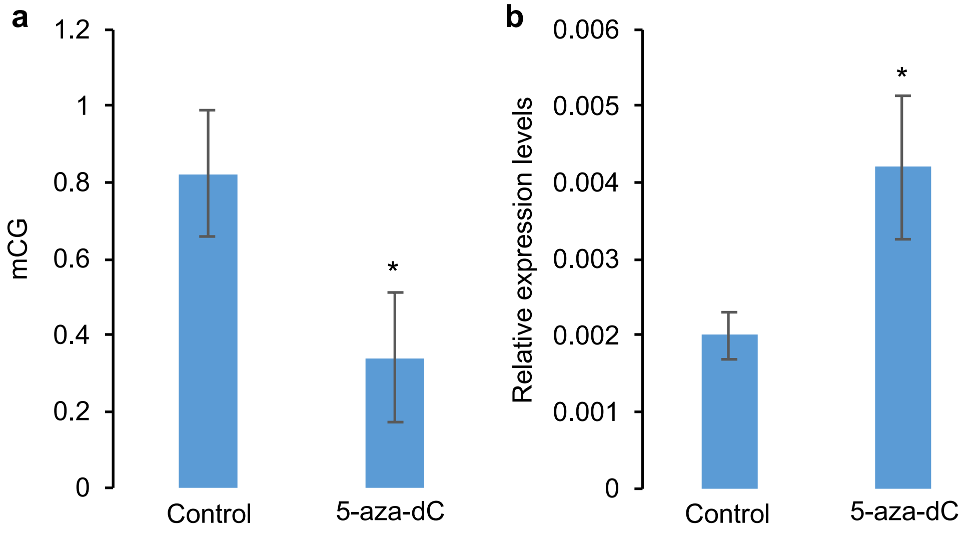

Figure S9: 5-aza-dC treatment increased expression levels of *COL2D* in wild *G. hirsutum* (TX2095). (**a**) DNA methylation levels in the boxed region of *COL2D* in Fig. 5e (D03: 32225460-32225724) were significantly decreased after 5-aza-dC treatment. (**b**) Relative expression levels of *COL2D* (normalized to *GhUBQ10)* was significantly increased after 5-aza-dC treatment.

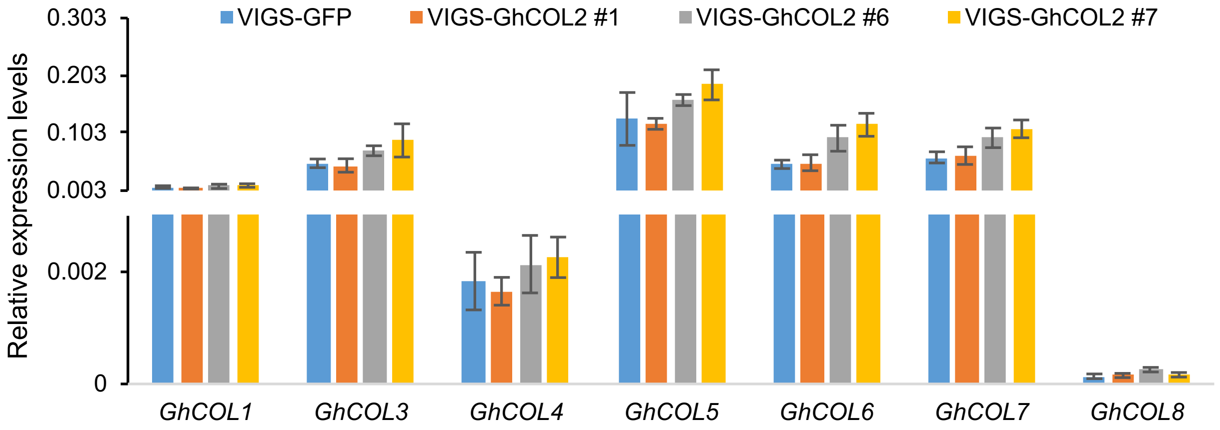

Figure S10: Other *GhCOLs* were not repressed in the VIGS lines of *GhCOL2*. Relative expression levels of *GhCOL1*, *GhCOL3*, *GhCOL4*, *GhCOL5*, *GhCOL6*, *GhCOL7*, and *GhCOL8* in the VIGS lines of *GhCOL2* and VIGS-GFP control**.** Expression levels of *GhCOLs* were normalized to *GhUBQ10.*

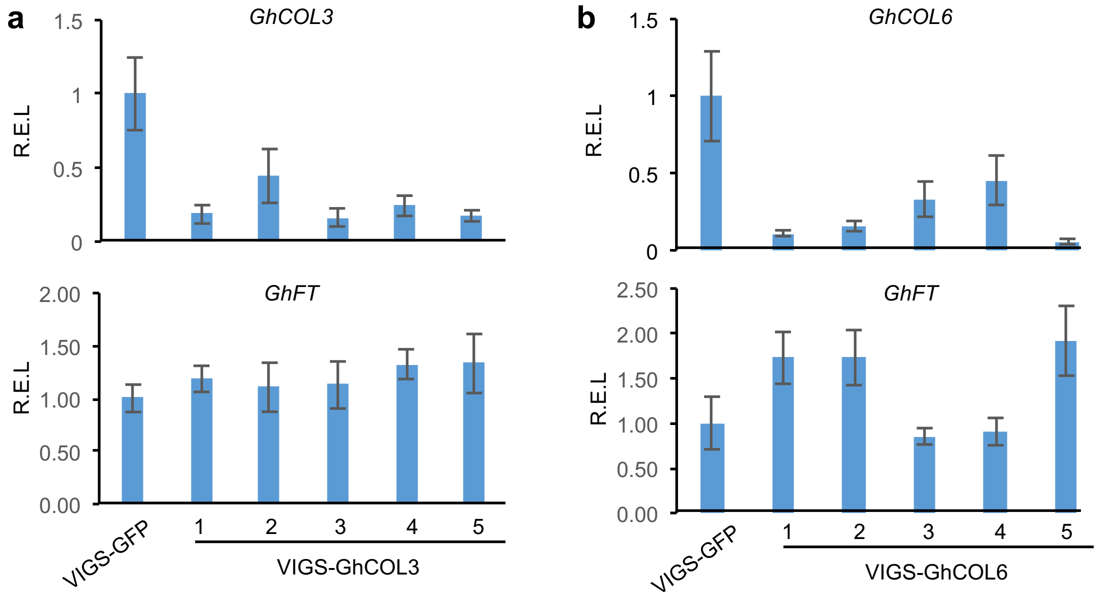

Figure S11: Repression of *GhCOL3* or *GhCOL*6 did not inhibit expression of *GhFT*. Relative expression levels (R.E.L) of *GhCOLs* and *GhFT* in five lines of virus-induced gene silencing (VIGS) each for *GhCOL3* (**a**) and *GhCOL6* (**b**). Expression levels of *GhCOLs* and *GhFT* in VIGS lines were normalized to those in the control (VIGS-GFP).
